# Establishment and application of information resource of mutant mice in RIKEN BioResource Research Center

**DOI:** 10.1186/s42826-020-00068-8

**Published:** 2021-01-18

**Authors:** Hiroshi Masuya, Daiki Usuda, Hatsumi Nakata, Naomi Yuhara, Keiko Kurihara, Yuri Namiki, Shigeru Iwase, Toyoyuki Takada, Nobuhiko Tanaka, Kenta Suzuki, Yuki Yamagata, Norio Kobayashi, Atsushi Yoshiki, Tatsuya Kushida

**Affiliations:** 1 Integrated Bioresource Information Division, RIKEN BioResource Research Center, 3-1-1 Koyadai, Tsukuba-shi, Ibaraki 305-0074 Japan; 2 Experimental Animal Division, BioResource Research Center, RIKEN, Tsukuba, Japan; 3 Laboratory for Developmental Dynamics, Center for Biosystems Dynamics Research, RIKEN, Kobe, Japan; 4 Data Knowledge Organization Unit, Head Office for Information Systems and Cybersecurity, RIKEN, Wako, Japan

**Keywords:** Bioresource, Mouse mutation information resource, Database, data integration, Semantic web, Ontology

## Abstract

Online databases are crucial infrastructures to facilitate the wide effective and efficient use of mouse mutant resources in life sciences. The number and types of mouse resources have been rapidly growing due to the development of genetic modification technology with associated information of genomic sequence and phenotypes. Therefore, data integration technologies to improve the findability, accessibility, interoperability, and reusability of mouse strain data becomes essential for mouse strain repositories. In 2020, the RIKEN BioResource Research Center released an integrated database of bioresources including, experimental mouse strains, *Arabidopsis thaliana* as a laboratory plant, cell lines, microorganisms, and genetic materials using Resource Description Framework-related technologies. The integrated database shows multiple advanced features for the dissemination of bioresource information. The current version of our online catalog of mouse strains which functions as a part of the integrated database of bioresources is available from search bars on the page of the Center (https://brc.riken.jp) and the Experimental Animal Division (https://mus.brc.riken.jp/) websites. The BioResource Research Center also released a genomic variation database of mouse strains established in Japan and Western Europe, MoG^+^ (https://molossinus.brc.riken.jp/mogplus/), and a database for phenotype-phenotype associations across the mouse phenome using data from the International Mouse Phenotyping Platform. In this review, we describe features of current version of databases related to mouse strain resources in RIKEN BioResource Research Center and discuss future views.

## Introduction

Mouse is one of the most important model organisms for studying biological phenomena in mammals. In particular, mice are prominent in human biology and disease research due to their genetic, genomic, phenotypic, and physiologic proximity to human. In the mammalian genetics research community, a number of genetically modified (GM) mice have been established using various artificial genetic engineering technologies such as transgenic, gene-targeting, and genome editing which are useful as in vivo models of human disease. Sharing of same mutant mouse strains across studies is essential to ensure reproducibility of experimental data. Online databases serve as an information resource for mouse mutant strains and play crucial a role providing: 1) a catalog of significant unique resources; and 2) a portal of genetic/phenotypic characteristics of the strains which is useful when designing experimental plans. In addition, global data sharing using data integration technology is key.

Modern biology has been accelerated by global sharing of biological data. The global community of mouse biology and genetics historically evolved via the accumulation of knowledge of mutant strains that helps transfer of knowledge among studies, hypothesis building, and experimental reproducibility by use of common mouse resources as experimental material which carries equivalent genetic characteristics. For example, Mouse Genome Informatics (MGI) greatly helps rapid accumulation of genetics/genomics knowledge [1, 2]. In cooperation with nomenclature standards for mouse genes and strains, MGI gives unique identifiers (UIDs) to data items in the database with name space of “MGI” such as “MGI:xxxxx” which is commonly used in multiple public databases in life science [3]. The description of genetic markers and mouse strains using a common identifier helps data access and integration and improves interoperability and reusability of data across databases. In mammalian research, authoritative data are provided by the MGI, the HUGO Gene Nomenclature Committee (HGNC), and the Rat Genome Database (RGD) with nomenclature activities for genes, alleles, and strains for each species [3–5]. Data from the National Center for Biotechnology Information (NCBI: https://www.ncbi.nlm.nih.gov) and Ensembl (https://ensembl.org) are also broadly used across species to provide identifiers [6, 7]. The International Mouse Strain Resource (IMSR), a combined catalog of worldwide mouse resources with direct access to repository sites holding resources [8], also records UIDs of mouse strains given by original repositories.

In the last 20 years, UID-based data sharing and integration have been performed on various types of data across databases. The Open Biomedical Ontology (OBO) Consortium provides sets of controlled vocabularies termed “ontologies” for complicated concepts such as gene function managed by UIDs (e.g. “GO:0000016” for “lactase activity”) developed with the aim of comprehensive annotation of biological information [9, 10].

In the MGI, ontologies for anatomical body parts (Mouse Anatomy Ontology: MA) [11] and phenotype (Mammalian Phenotype Ontology: MP) [12] were developed for annotation of characteristics of mutant strains. Using these ontologies, completeness of searching has been significantly improved in MGI and RGD [12, 13]. Ontology also helps representing meaning of terms or concepts by its graph structure composed of links among UIDs. The development of methodologies for computational comparison between phenotype ontology terms allows linking of phenotype records of model animals to diseases which are annotated with Human Phenotype Ontology (HPO) automatically [14–19]. Therefore, description of mouse strains themselves and their metadata (e.g. genetic and phenotypic features) using UID-based data items such as ontologies is remarkably beneficial for strain repositories to represent how mouse strains can be useful for disease studies. In addition, Research Resource Identifiers (RRIDs) are proposed to help researchers citing bioresources used in their study [20] including, antibodies, model organisms, and tools (i.e. software and databases).

**Fig. 1:**
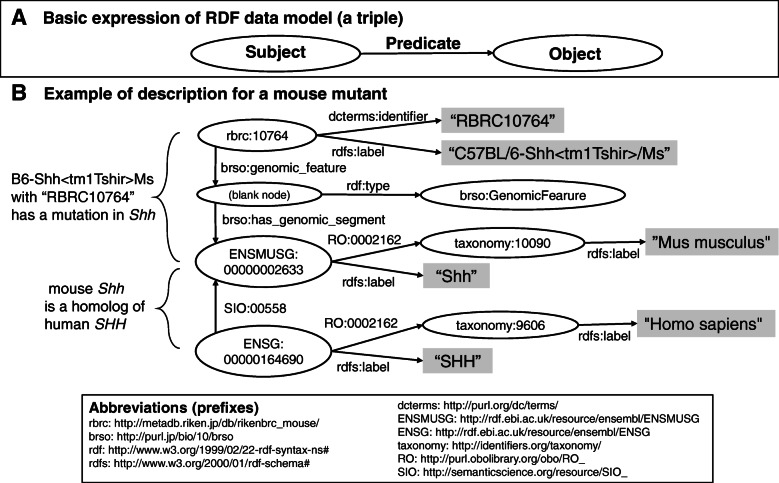
Basic semantic expression of RDF data model. **a** RDF data model is based on the simple expression in a “triple” which is a set of three entities, subject, predicate and object to describe resources in the WWW. Subjects and predicates are addressed in URIs. An object is addressed in an URI or text. **b** An example of RDF data model forming a knowledge graph describing a mouse mutant represented in multiple triples. Each URIs are represented with abbreviations (prefixes) listed in the box at the bottom part. Note that most URIs were defined outside RIKEN (as common vocabularies). Upper parts of the graph describe rbrc:10764 named as “C57BL/6-Shh < tm1Tshir>/Ms” with identifier “RBRC10764” has a mutation in the *Shh gene* of *Mus musculus*. The blank node is an anonymous record representing a genomic feature of C57BL/6-Shh < tm1Tshir>/Ms. (rbrc:10764). Lower part describes *Shh* is a homolog of the *SHH* gene of *Homo sapiens*

There is a growing concern regarding the capacity of computational systems as a result of the increase in volume, complexity, and creation speed of data. The “semantic web” offers a series of methods and technologies to develop extensions of the current World Wide Web (WWW) in which information is given well-defined meanings and integration [21]. These technologies include the Resource Description Framework (RDF) [22] which is a framework to provide a formal description of concepts, terms, and relationships within a given knowledge of domains recommended by the World Wide Web Consortium (W3C). In the RDF, informational resources on the Web are named by unique resource identifiers (URIs) which work as UID on the WWW. URIs in RDF are usually described as uniform resource locators (URL) on the Web. URL helps to show where you need to access to obtain more information about the resource. The expressiveness allows RDF to define controlled vocabularies with exact relationships to other informational resources. RDF represents a powerful data model for data federation and interoperability across various datasets. The data model of RDF, which forms knowledge graphs composed of interlinking URIs, provides interoperability between applications that exchange machine-readable information on the Web (Fig. 1). Currently, multiple database management systems that store and process RDF data (often termed as “triples” because RDF data model is described in expression of the subject-predicate-object: see Fig. 1a) are called RDF stores or triple stores as introduced in https://db-engines.com/en/article/RDF+Stores. The common query language of RDF, SPARQL,^1^ which allows for a query to consist of triple patterns, conjunctions, disjunctions, and optional patterns is openly provided and can act as a common Application Programing Interface (API)^2^ of RDF stores on the Web. Furthermore, the “federated query” extension of SPARQL^3^ allows executing queries distributed over different SPARQL endpoints. Using this extension, users can easily merge data distributed across the Web. From these features, RDF-based data integration is considered to be one of the basic strategies for making research results available through the Web.

In 2016, FORCE11, a community of scholars, librarians, archivists, publishers, and research funders that has arisen organically to help facilitate the change toward improved knowledge creation and sharing formulated FAIR Data Principles (https://www.force11.org/group/fairgroup/fairprinciples)^4^ act as foundational guidelines to improve the findability, accessibility, interoperability, and reuse of digital assets. It is suggested that there is an urgent need to improve the infrastructure that supports the reuse of academic data according to the FAIR principle [23]. Hence, data integration technology becomes non-negligible for repositories of bioresource due to efficient use of resources in the scientific community.

The RIKEN BioResource Research Center (BRC), a global not-for-profit public institution providing biological materials, technical services, and educational programs, has collected, preserved, and provided experimental mice, *Arabidopsis thaliana* as a laboratory plant, cell lines derived from humans and animals, microorganisms, and associated genetic materials. Mice are one of the major resources in RIKEN BRC. In 2019, RIKEN BRC collected over 8800 mouse strains including inbred, spontaneous mutant, recombinant inbred, consomic, transgenic, knockout, wild-derived, and genome editing [24]. RIKEN BRC develops its informatics activity to ensure the wide dissemination of bioresource data and contributes to develop novel utilities and create new “values” and facilitate wide, effective and efficient use of bioresources for R&D in science and industry. The core activities of RIKEN BRC’s informatics include: 1) data integration and standardization aiming wider dissemination of bioresource data and additional metadata for biological properties of bioresources such as genome, phenotype, and conceivable research use in collaboration with other biological databases and medical data; 2) improvement of homepage contents to provide user-friendly interface for biologists; and 3) big data analysis aiming to facilitate novel utilities with discoveries of hidden values of bioresources. Here, we review the development of mouse strain resources in RIKEN BRC as the basic infrastructure for promotion of the life science.

## Main text

### Development of an information resource of mutant mice using data integration technologies in RIKEN BRC

#### Bioresource online catalog

Aiming to meet the needs summarized in the FAIR principles toward the wider dissemination of bioresource information and thereby adding value to bioresources, RIKEN BRC has been applying data integration technologies based on the RDF-related technologies for database of bioresources [25, 26]. There are number of advantages when using RDF for bioresource database. Firstly, the RDF data model forming graphs (instead of tables in relational database) is flexible. New data is easily added by adding a new column and updating the schema. This feature is suitable for bioresource databases to add a variety of information or data of biological characteristics to bioresources. Since bioresource usage is greatly influenced by scientific trends, it is extremely important that they can be easily expanded according to the needs. Secondly, it is easy to integrate bioresource information with other RDF-based public datasets in life science. In the bioresource database, description of genetic and phenotypic characteristics is essential to promote the use of bioresources. RDF provides a simple way of description of biological characteristics referring URIs of public data of genetic markers which involves gene and alleles and ontology terms of phenotypic characteristics bioresources. Thirdly, RDF provides fundamental infrastructure of data dissemination on the Web. If the RDF-based statement of bioresources is openly available on the Web, anyone can refer an URI of a bioresource for description of an experimental result, allowing the establishment of the exact relationship of the bioresource and the experiment on the Web. This helps to ensure reproducibility of experimental data.

**Fig. 2:**
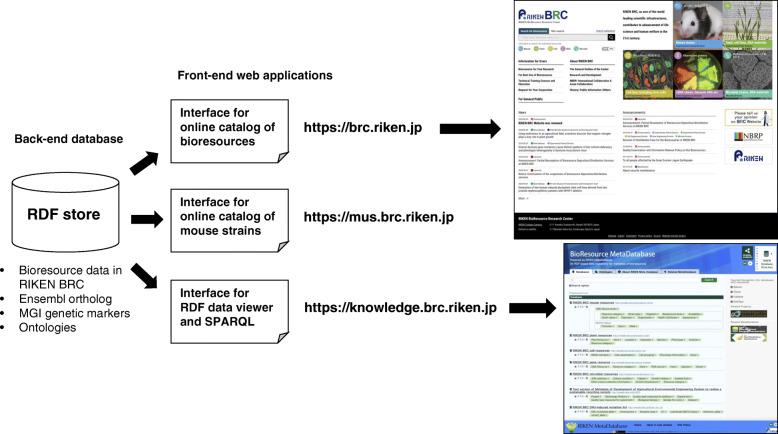
Overview of bioresource integrated database in RIKEN BRC. Left panel shows composition of the bioresource integrated database. The integrated database for online catalog of bioresources in RIKEN BRC is composed of a back-end database and the front-end web applications. Three different applications (online catalogs of whole bioresources in RIKEN BRC, mouse and RDF data viewer and SPARQL search) are hosted by the back-end database. Right panel shows web interfaces of https://brc.riken.jp and https://knowledge.brc.riken.jp. Interface of https://mus.brc.riken.jp is shown in Fig. 2

The current version of online catalog of mouse strains in RIKEN BRC is implemented as part of the integrated database of whole five bioresources in RIKEN BRC including, experimental mice, *Arabidopsis thaliana*, cell lines, microorganisms, and genetic materials. The database is composed of a back-end database engine called “RDF store” and a front-end software program to generate dynamic Web-based GUI for retrieval to RDF store showing search results of mouse strains (Fig. 2).

In the back-end RDF store, implemented with Virtuoso (OpenLink Software Inc.), basic data of bioresources in RIKEN BRC including mouse strains, mouse gene and allele data imported from MGI [2] and RDF version of Ensembl (https://www.ebi.ac.uk/rdf/documentation/ensembl/) [27] are stored. In the RIKEN BRC, mouse strains are managed with UIDs composed of “RBRC” (meaning RIKEN BRC) prefix and strain number. For the generation of RDF versions of mouse strain data, the URI of mouse strain records are generated referring RBRC-number. Basic data of mouse strains contains strain’s UID, name, synonym(s) for name, back ground strain, description for strain development and phenotype, link to health report, gene mutation (allele), specific term and conditions for use of the strain, status of preservation, and publication among others. Genes and alleles mutant strains have been described with common UIDs and URIs. Data imported from MGI and Ensembl are used to allow searching by gene name, synonyms, and name of homologs.

**Fig. 3:**
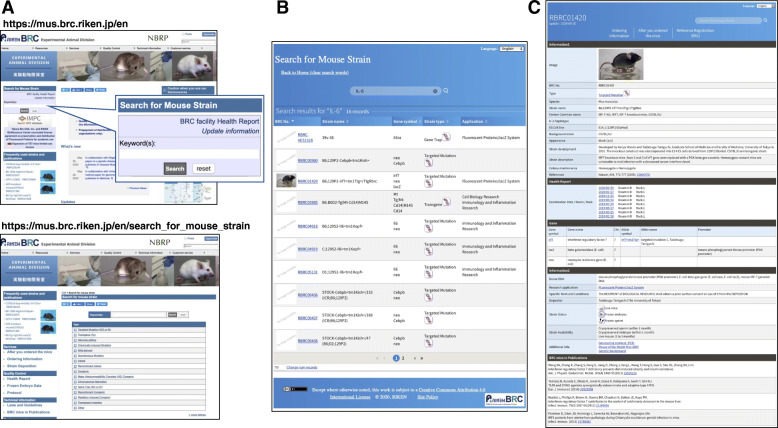
Overview of mouse strain search. **a** Search bars of mouse strain in RIKEN BRC are represented at the top page of Experimental Animal Division’s Page (https://mus.brc.riken.jp: upper panel) and the search page (https://mus.brc.riken.jp/en/search_for_mouse_strain: lower panel). **b** An example of search result for mouse strains that have homologs related to the keyword entered are listed. **c** Detailed information page for a mouse strain including health reports, alleles and publications. The detailed page is linked to ordering information page for mouse strains

The Web interface of mouse strain search is integrated in the RIKEN BRC (https://brc.riken.jp) and the Experimental Animal Division (https://mus.brc.riken.jp/) websites (top-page of the both websites). Suggestions of gene and strain names are listed according to the characters entered by the user dynamically. The search result is represented as a summarized list of strains that meet the search condition. The detailed information page of a mouse strain is linked from the summarized list page (Fig. 3).

In addition to the mouse strain search interface, users can directly access to mouse strain data via another Web interface (https://knowledge.brc.riken.jp/bioresource/db/xsearch_animal) that is a direct interface of the back-end RDF store operated by “Bioresource MetaDatabase” (https://knowledge.brc.riken.jp/) which is an instance of the RIKEN MetaDatabase software application [26]. The Bioresource MetaDatabase provides a simple common view of RDF, download function of RDF data, and a SPARQL endpoint that accepts SPARQL queries and returns results. Using the SPARQL query language, users can output data in a user-specified format from the endpoint. In particular, using federated query syntax of SPARQL, users can obtain combined results between mouse strains and related biological information such as genome, proteins, and diseases which are hosted in other SPARQL endpoints such as the EBI RDF platform (https://www.ebi.ac.uk/rdf/) [27] and DisGeNET (https://www.disgenet.org) [28]. Mouse strain data in RIKEN BRC is regularly uploaded and available at IMSR which is an international portal of mouse strain repositories (http://www.findmice.org) [8]. Assignment of RRID for mouse strains in RIKEN BRC is already done via the IMSR database (e.g. RRID: IMSR_RBRC09538).

#### Genetic variation database “MoG^+^”

**Fig. 4:**
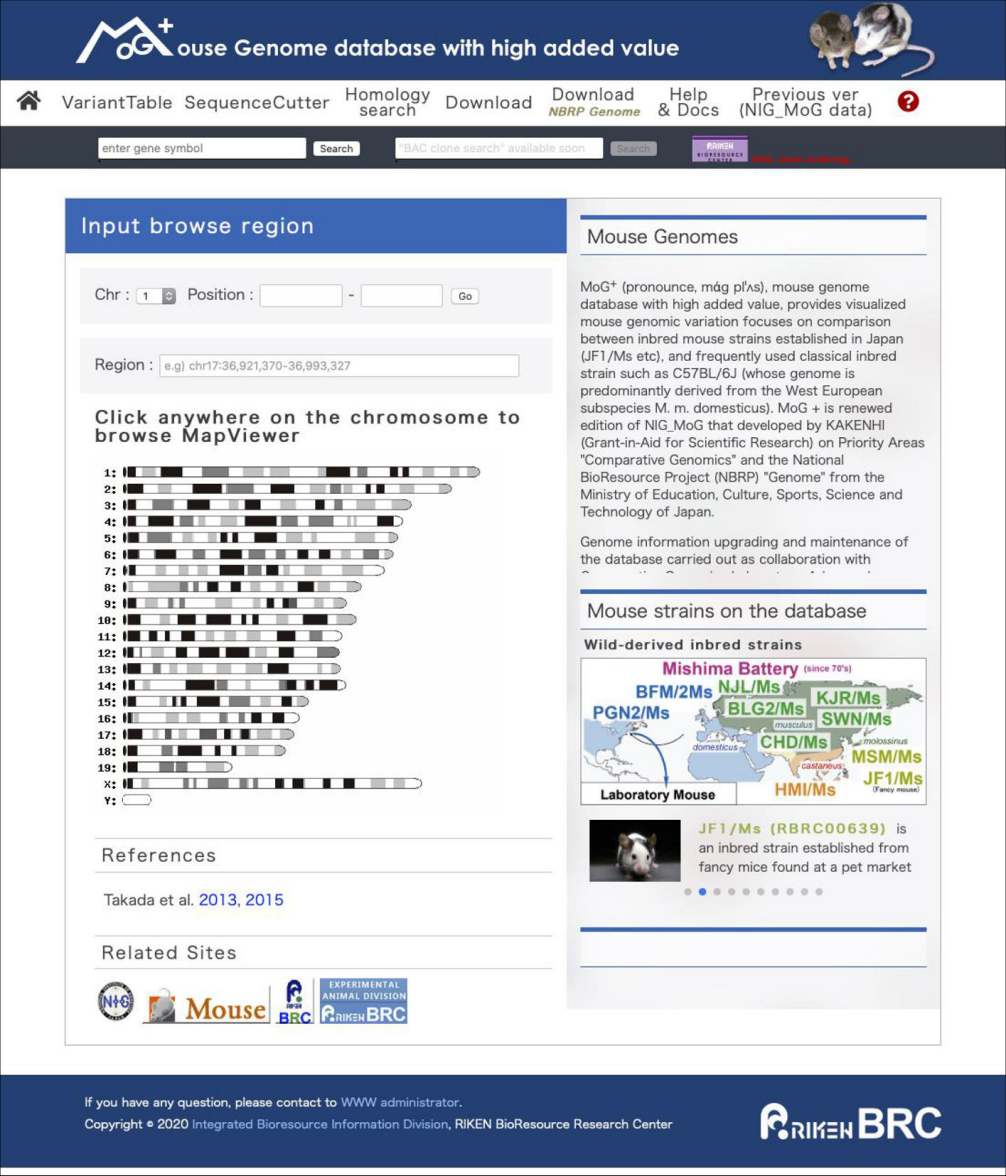
The top page of MoG+ database (https://molossinus.brc.riken.jp). Detailed explanation of this web application is available at the tutorial page (https://github.com/ttakada1/MoGplus_tutorial_2020/wiki)

Studies of genetic and genomic variation are essential to understand the genetic contribution and pathogenesis of diseases in human. As a mammalian model of genetic variation of human, genetically diverse mouse inbred strains provide multiple important biological insights [29]. To provide mouse genetic variation compared with inbred mouse strains established in Japan (JF1/Ms. etc), and frequently used classical inbred strain such as C57BL/6 J (whose genome is predominantly derived from the West European subspecies *M. m. domesticus*), RIKEN BRC operates a mouse genetic variation database named “MoG^+^” (pronounced mάg plˈʌs) which is an expanded version of a genome database, NIG_MoG, transferred from the National Institute of Genetics [30, 31] (Fig. 4). MoG^+^ takes over polymorphism information on nucleotide substitutions and short indels, focusing on the protein-coding genes of the two *M. m. molossinus*-derived strains with respect to those of B6 and other representative classical inbred mouse strains based on the results of re-sequencing experiments. The GUI of MoG^+^ provides gene search, BAC clones deposited in RIKEN BRC, and genomic region. Search results are visualized as a map view, sequence alignment, details of coding region and gene feature, and detailed information of BAC.

#### Contribution to the international mouse Phenotyping Consortium and provision of an advanced reference data set

The International Mouse Phenotyping Consortium (IMPC) was established in 2011 [32] expanding international collaborative networks in phenotyping knockout mice, with the goal to publish an Encyclopedia of the Mammalian Genome Function. RIKEN BRC has committed to this effort. These collaborative activities will greatly contribute to delineate the biological gene function, a better understanding of diseases, drug discovery and development as well as prediction of potential side-effects early in the drug discovery process, and moreover, deeper insight into sophisticated biological functions. Phenotype data analyzed in RIKEN BRC by the comprehensive mouse phenotyping platform, Japan Mouse Clinic [33], is regularly transported to the data coordination center of IMPC through the data conversion pipeline which transforms raw experimental data to the standardized format in IMPC. Phenotype data analyzed in RIKEN BRC is available at the IMPC website (http://www.mousephenotype.org) with the result of automatic phenotype annotation by MP ontology. The IMPC dataset represents the largest and most reliable phenotype data using standardized genetic background, experimental methods [34] analysis workflows, and control strategies [35]. The mouse strains used in the phenotype analyses are available from RIKEN BRC.

**Fig. 5:**
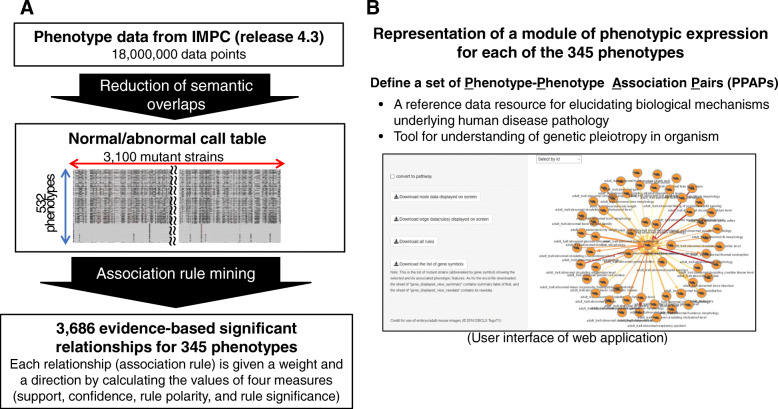
Overview of the strategy for phenome-wide association study in RIKEN BRC. **a** Overview of generation of high-quality association rules using phenotype data from IMPC. We used IMPC release 4.3 dataset contains approximately 18,000,000 data points for 2050 parameters. We made a call table of normal/abnormal phenotypes with 532 phenotypes for 3100 mutant strains (genes) by the data processing of ontology-based reduction of semantic overlaps for parameters. 3686 relationships (association rules) were obtained by the association rule mining analysis. **b** For evaluation and understanding of the relationships, we defined a set of PPAPs as a module of phenotypic expression for each of the 345 phenotypes. Upper panel shows a schematic diagram of PPAP in which a phenotype associated to other phenotype with statistical weight of association. Lower panel shows data representation in the web application (https://brc-riken.shinyapps.io/phenotypic_associations_across_the_mouse_phenome/) in which users can select, search and download any relationships. See Tanaka et al. (2020) [37] for details

To provide reliable, weighted phenotype-phenotype relationships as a reference data resource useful to detailed evaluation of the mouse mutant resources, RIKEN BRC worked out an association rule mining of a dataset consisting of only binary (normal and abnormal phenotypes) data to determine relationships among phenotypes with IMPC data which is a bias-minimized comprehensive data of mouse phenotypes. As a result, a set of phenotype-phenotype association pairs (PPAPs) as a module of phenotypic expression for each of the 345 phenotypes were defined [36, 37]. By analyzing each PPAP, phenotype sub-networks consisting of the phenotypes and distinct biological systems were also defined. Hierarchical clustering based on phenotype similarities among the 345 PPAPs revealed seven community types within a putative phenome-wide association network (Fig. 5). These mouse phenome-wide phenotype-phenotype association data reveal general principles of relationships among mammalian phenotypes and provide a reference resource for biomedical analyses. To promote use of the association data, we developed and published web-application tools available at: https://brc-riken.shinyapps.io/phenotypic_associations_across_the_mouse_phenome/ and https://brc-riken.shinyapps.io/associations_between_biological_systems/.

#### Future directions

Above, we overviewed newly released information resources of mouse mutant strains in RIKEN BRC in 2020. Currently, mouse strains including GM mice are expected to contribute more and more as animal models of human diseases. Thus, the requirement for mouse information resources is becoming more sophisticated to quickly respond to the needs of scientific communities. This trend has become more pronounced during the 2020 coronavirus crisis. Aiming to promote the advanced use and to help expand the use of mouse mutant resources, RIKEN BRC continuously supports the development of information technologies on data integration, implementation of advanced data searching system, and data analysis.

Further collaboration with RRID is a non-negligible issue for bioresource repositories. Although mouse strains and cultured cells in RIKEN BRC are already registered in the RRID database, we need to promote RRID for plant, microbes, and genetic material.

Further data integration across different datasets, online catalogs, genome variation databases and datasets produced from advanced mathematical methods such as the phenotype-phenotype association described above is also one of the major issues. One possible way to achieve this is to apply RDF-related technology to datasets such as MoG^+^ and advanced data analysis methods and dataset derived from the data analysis. MoG^+^ is planned to enhance through the collaboration with human genetic variation databases such as TogoVar (https://togovar.biosciencedbc.jp/) which collects and organizes human genomic variants in the Japanese population assorted by disease information [38] to provide mouse-human orthologous relationships of genetic variants which are valuable for modeling of human disease. Generation of an RDF version of variation data such as Variant Call Format (VCF) may be also valuable for global data integration of genetic variation and bioresource information.

The online bioresource catalog in RIKEN BRC is planned to have enhanced search functions with better data integration with genomics, genetics, and phenotype data. The gene-based search of bioresources will be enhanced referring another dataset than Ensembl. We currently plan to introduce an RDF version of the Orthologous Matrix (OMA: https://omabrowser.org/oma/home/) database [39] to our RDF store. The OMA database is a comprehensive resource to relate genes across a wide range of species generated by the OMA inference algorithm to identify orthologous pairs and hierarchical orthologous groups in which distinction between orthologs (genes or genomic segments in different species evolving from a common ancestral sequence) and paralogs (copies of genes or genomic segments created by a duplication events within the same species) is ensured. Improvement of the accuracy of gene search function to avoid listing too many genes with paralogous relationship is expected using OMA, in which orthologous and paralogous relationship are implemented as different relationships.

It is also planned the implementation of mouse strain search with vocabularies of phenotype and human disease. As described above, providing phenotype information is one of the most important functions required for the mouse information resource which is a serious weakness in the current version of RIKEN BRC mouse strain database. To address this, a curation of mouse strain phenotypes using MP ontology [12] is ongoing in RIKEN BRC. We also plan to introduce phenotype-related ontologies (MP, MA, HPO) which are available as Web Ontology Language (OWL: an RDF-compatible language for description of ontology) and data library to relate ontology terms of mouse phenotypes to human diseases [14–19] to our RDF store. In addition to these datasets, DisGenNET provides gene-disease relationships [28]. Using these datasets in our RDF store or via the federated query function that allows data inquiry across different RDF stores, we plan to enhance the search function of the bioresource database to suggest mouse strains which are genetically or phenotypically related to human diseases. With these enhancements, RDF-based technology provides a cost-effective way of development and implementation.

Provision of advanced mathematical methods to analyze big-data is one of the most effective ways to add value to bioresources discovery of novel biological functions or principles of life systems. In addition to phenotype-phenotype associations, we also work in the development of a novel mathematical method termed “Energy landscape analysis” to visualize energy states or stabilities using omics data of microflora or transcriptome of environments or organisms [40]. This methodology is expected to be useful for controlling the biological state of soil, intestine, and cell differentiation which are composed of multiple factors with intricate interactions. Providing an RDF version of resultant data showing direct links to bioresources as materials of the experiment will significantly contribute to add value to bioresources. Sharing of workflow for computational data analysis using container virtualization technologies is another issue to consider [41].

There is a lot of information that should be shared to improve the value of bioresources. The Asian Mouse Mutagenesis Resource Association (AMMRA) is a collaborative group for the development, archiving/distribution, coordination of phenotyping, and informatics of mutant mice in Asia and Australia developed for sharing expertise, mouse modelling resources, and technology developments for the rapid assessment of variants through model-on-demand programs, biorepositories, and phenotyping clinics, to researchers and clinicians. At the AMMRA website (http://ammra.info/), we share information of mouse resources as well as the training of technicians, researchers, and clinicians in mouse genetics and resources. Further international collaboration in bioinformatics and data integration among repositories may greatly contribute to facilitate advanced infrastructure for life science.

## Conclusion

The infrastructure of science to ensure reproducibility and application of data integration technology is non-negligible for online data resources of mouse mutant repositories. In 2020, RIKEN BRC released three new databases of the integrated online catalog for bioresources including mouse strains, mouse genome variation database, and a database for phenotype-phenotype association. With the integration of other RDF resources such as Ensembl, the database of mouse strain provides better function of gene-based search. To ensure the addition of further value to the mouse strain resource as a common experimental material, further enhancement, integration, and collaboration of mouse information resources are planned to provide better use of the information with richness, organization and standardization.

## Data Availability

Datasets described in this review are available at https://knowledge.brc.riken.jp, https://molossinus.brc.riken.jp/pub/, https://brc-riken.shinyapps.io/phenotypic_associations_across_the_mouse_phenome/ and https://brc-riken.shinyapps.io/associations_between_biological_systems/

## Abbreviation

SPARQL: A recursive acronym for SPARQL Protocol and RDF Query Language (https://en.wikipedia.org/wiki/SPARQL#cite_note-2)

## Footnotes

1 SPARQL SPARQL (a recursive acronym for “SPARQL Protocol and RDF Query Language”) is a query language for retrieval and manipulation of data stored in RDF format. SPARQL has been standardized by the RDF Data Access Working Group (DAWG) in WWWC.

2 API Application Programming Interface (API) is a computing interface used by software components to exchange information with each other. The important role of the API is to provide programmatic operation method (interface) for a software application from the other software applications. APIs allow developers to manipulate remote resources through protocols, specific standards for communication that allow different technologies to work together.

3 Federated query extension of SPARQL Federated search retrieves information from multiple data sources through a search engine by the process that single query request is distributed to multiple databases. In SPARQL, federated query protocol allows to build a query to be distributed to multiple SPARQL endpoints (services that accept SPARQL queries and return results) and results gathered.

4 FAIR data principles The FAIR Data Principles are proposed by a voluntary group of researchers, librarians and publishers called FORCE 11 (Future of Research Communications and e-Scholarship) aiming creation and sharing of knowledge in 2014. The Principles provides a set of guiding principles making data findable, accessible, interoperable and reusable to provide guidance for scientific data management and stewardship to promote maximum use of research data. Capacity of computational systems to find, access, interoperate, and reuse data with minimal human intervention are extremely important elements for the Principles because humans increasingly rely on computational support to deal with huge data. Practical hints on how they could be implemented is explained at http://fairprinciples.org/.
